# Supplementary material for: Insulin Predicts Methotrexate Response by Affecting the Transcription of Methotrexate Target Genes in the Treatment-Naive Rheumatoid Arthritis
Source: Cells. 2025 Jun 24;14(13):964. doi: 10.3390/cells14130964 (PMC12249082; doi:10.3390/cells14130964)
Supplement: Supplementary file 1 [file cells-14-00964-s001.zip › cells-3671945-supplementary.pdf]

Supplementary figure S1.

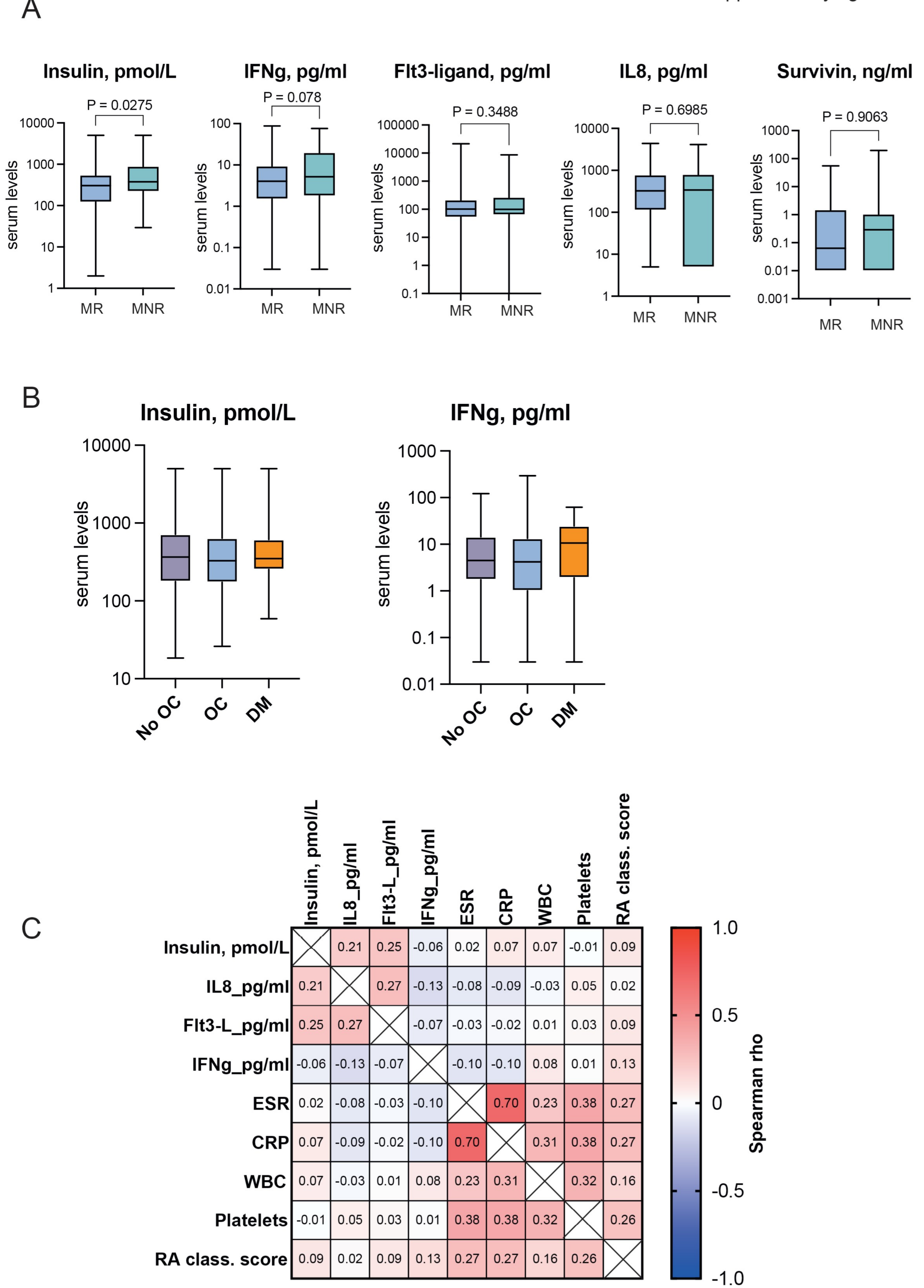

Supplementary Table S1. Clinical characteristics of the MTX-treated patients grouped by the year of enrollment.

	Cohort 1<br>2012-2013<br>(n=93)	Cohort 2<br>2018-2019<br>(n=164)
MTX treated, n (%)	60 (64.5)	112 (68.3)
Female, n (%)	41 (68.3)	75 (67.0)
Age, y	53.9 (26-83)	56.2 (22-88)
Smokers, n (%)	26 (43.3)	43 (38.4)
Diabetes mellitus, n	7	10
RA antibodies, positive	42 (70)	60 (53.6)
RA classification score*<br>≥6 points, n (%)	6.71 (10-2)<br>45 (75)	6.08 (10-2)<br>72 (64.3)
Swollen joints, n	6.25 (1-17)	5.74 (1-22)
**At 1 year**<br>MTX, n (%)<br>MTX dose, mg/week*<br>Other DMARDs, n<br>Biologics, n<br>Tested DMARDs, n	 50 (83.3)<br>19.9 (7.5-30)<br>11 (18.3)<br>19 (31.7)<br>1.55 (0-3)	 90 (80.4)<br>18.73 (10-25)<br>11 (9.8)<br>27 (24.1)<br>1.65 (0-3)
Oral corticosteroids<br>at 1st visit, n (%)<br>at 1 year, n (%)	 34 (56.7)<br>23 (38.3)	 75 (67.0)<br>31 (27.7)
Remission at 1y, n (%)	24 (40)	48 (42.9)

*Mean(min-max). P-values between the cohorts were calculated by Mann-Whitney U test or by chi-square mid-exact.

Supplementary Table S2: Detailed steps in development of the MTX response predictive model.

Dependent variable: MTX responder=1, MTX nonresponder=0
n=172, 53.4% MTX responder

Model 1. RA class. score, age, Female gender, current smoker, Oral Corticosteroids, DM.

Parameter estimates	Variable	beta Estimate	Standard error	Lower 95 % conf, lim	Upper 95 % conf, lim	OR Estimate	Lower 95% conf, limit (p	Upper 95 % conf, lim	|Z|	P value	P value summary	Covariance, VIF	R2 with other variables
β0	Intercept	-0,01477	0,8026	-1,589	1,576	0,9853	0,2041	4,835	0,0184	0,9853	ns		
β1	RA classification score	-0,3195	0,09058	-0,5047	-0,1476	0,7265	0,6037	0,8628	3,527	0,0004	***	1,132	0,1162
β2	Age, y	0,04348	0,0122	0,02036	0,06841	1,044	1,021	1,071	3,564	0,0004	***	1,066	0,06173
β3	Female gender	0,2657	0,3688	-0,455	0,9965	1,304	0,6345	2,709	0,7206	0,4712	ns	1,058	0,05467
β4	Current smoking	0,4985	0,3571	-0,1935	1,212	1,646	0,8241	3,359	1,396	0,1627	ns	1,059	0,05545
β5	Oral corticosteroids	-0,8737	0,368	-1,613	-0,1639	0,4174	0,1994	0,8488	2,374	0,0176	*	1,068	0,06363
β6	Diabetes mellitus	-0,2806	0,5778	-1,415	0,8789	0,7554	0,2429	2,408	0,4856	0,6272	ns	1,033	0,03181

Area under the ROC curve	
Area	0,7272
Std. Error	0,03815
95% confider	0,6525 to 0,8020
P value	<0,0001

predictive power (%)	
Negative predicti	64,86
Positive predictiv	67,35

Model 2. Swollen joints, nAB, WBC, Platelets, CRP, ESR

Parameter estimates	Variable	beta Estimate	Standard error	Lower 95 % conf, lim	Upper 95 % conf, lim	OR Estimate	Lower 95% conf, limit (p	Upper 95 % conf, lim	|Z|	P value	P value summary	Covariance, VIF	R2 with other variables
β0	Intercept	2,191	0,75	0,7746	3,737	8,944	2,17	41,97	2,921	0,0035	**		
β1	Swollen joints, n	-0,08188	0,03715	-0,1567	-0,01	0,9214	0,855	0,99	2,204	0,0275	*	1,082	0,07555
β2	RA-ab, 0, any, both	-0,4478	0,1793	-0,8049	-0,09973	0,639	0,4471	0,9051	2,497	0,0125	*	1,034	0,03303
β3	ESR	-0,006527	0,01033	-0,02708	0,0137	0,9935	0,9733	1,014	0,6319	0,5275	ns	2,097	0,5231
β4	CRP	0,00491	0,007784	-0,01004	0,02098	1,005	0,99	1,021	0,6308	0,5282	ns	2,282	0,5618
β5	WBC	-0,02267	0,07499	-0,1721	0,1239	0,9776	0,8419	1,132	0,3023	0,7625	ns	1,266	0,21
β6	Platelets	-0,00242	0,001979	-0,006451	0,001386	0,9976	0,9936	1,001	1,223	0,2215	ns	1,707	0,414

Area under the ROC curve	
Area	0,6802
Std. Error	0,04037
95% confider	0,6010 to 0,7593
P value	<0,0001

predictive power (%)	
Negative predicti	63,24
Positive predictiv	64,42

Model 3. Insulin, IFNg, IL8, Survivin, Flt3-ligand

Parameter estimates	Variable	beta Estimate	Standard error	Lower 95 % conf, lim	Upper 95 % conf, lim	OR Estimate	Lower 95% conf, limit (p	Upper 95 % conf, lim	|Z|	P value	P value summary	Covariance, VIF	R2 with other variables
β0	Intercept	0,1645	0,2345	-0,299	0,6244	1,179	0,7416	1,867	0,7016	0,4829	ns		
β1	Insulin_pmol/L	0,0007822	0,0002455	0,000344	0,001315	1,001	1	1,001	3,186	0,0014	**	1,354	0,2613
β2	IFNg_pg/ml	-0,03842	0,01275	-0,06543	-0,01508	0,9623	0,9367	0,985	3,013	0,0026	**	1,118	0,1055
β3	Survivin_ng/ml	-0,004321	0,009281	-0,02717	0,01333	0,9957	0,9732	1,013	0,4656	0,6415	ns	1,013	0,01264
β4	IL8_pg/ml	-0,00008338	0,0001945	-0,000475	0,000301	0,9999	0,9995	1	0,4286	0,6682	ns	1,259	0,2055
β5	Flt3-ligand_pg/ml	-0,00007314	0,0001146	-0,000278	0,000199	0,9999	0,9997	1	0,6385	0,5232	ns	1,332	0,2495

Area under the ROC curve	
Area	0,6486
Std. Error	0,04155
95% confider	0,5672 to 0,7301
P value	0,0008

predictive power (%)	
Negative predicti	58,18
Positive predictiv	58,97

Model 4. RA class. score, age, Female gender, Insulin, IFNg

Parameter estimates	Variable	beta Estimate	Standard error	Lower 95 % conf, lim	Upper 95 % conf, lim	OR Estimate	Lower 95% conf, limit (p	Upper 95 % conf, lim	|Z|	P value	P value summary	Covariance, VIF	R2 with other variables
β0	Intercept	-0,06684	0,8305	-1,705	1,571	0,9353	0,1817	4,812	0,08048	0,9359	ns		
β1	RA classification score	-0,3764	0,09745	-0,5772	-0,1931	0,6863	0,5615	0,8244	3,863	0,0001	***	1,073	0,0682
β2	Age, y	0,03904	0,01218	0,01591	0,06392	1,04	1,016	1,066	3,205	0,0014	**	1,043	0,04078
β3	Female gender	0,3614	0,3872	-0,3923	1,133	1,435	0,6755	3,104	0,9334	0,3506	ns	1,041	0,0394
β4	Insulin_pmol/L	0,000874	0,0002596	0,000422	0,00145	1,001	1	1,001	3,367	0,0008	***	1,085	0,07824
β5	IFNg_pg/ml	-0,03311	0,01307	-0,06104	-0,009164	0,9674	0,9408	0,9909	2,533	0,0113	*	1,102	0,09289

Area under the ROC curve	
Area	0,7625
Std. Error	0,03563
95% confider	0,6927 to 0,8323
P value	<0,0001

predictive power (%)	
Negative predicti	68,49
Positive predictiv	69,7
